# Acquired amphotericin B resistance attributed to a mutated *ERG3* in *Candidozyma auris*

**DOI:** 10.1128/aac.00601-25

**Published:** 2025-09-22

**Authors:** Lauryn Massic, Laura A. Doorley, Sarah J. Jones, Irene Richardson, Danielle Denise Siao, Lauren Siao, Philip Dykema, Chi Hua, Emily Schneider, Christina A. Cuomo, P. David Rogers, Stephanie Van Hooser, Josie E. Parker, Steven L. Kelly, David Hess, Jeffrey M. Rybak, Mark Pandori

**Affiliations:** 1 Nevada State Public Health Laboratory 736803, Reno, Nevada, USA; 2 University of Nevada Reno School of Medicine 12290, Reno, Nevada, USA; 3 St Jude Children's Research Hospital 5417, Memphis, Tennessee, USA; 4 Washington State Department of Health 11153, Olympia, Washington, USA; 5 Brown University Division of Biology and Medicine 174279, Providence, Rhode Island, USA; 6 Broad Institute 33577, Cambridge, Massachusetts, USA; 7 Cardiff University Schools of Biosciences 2112, Cardiff, Wales, United Kingdom; 8 Swansea University Medicine Health and Life Science 639919, Swansea, Wales, United Kingdom; University of Iowa, Iowa City, Iowa, USA

**Keywords:** microbial, public health, genetics, ERG3, amphotericin B, multidrug resistance, *Candida auris*

## Abstract

First identified in 2009, *Candidozyma auris* (formerly *Candida auris*) is an emerging multidrug-resistant fungus that can cause invasive infections with a crude mortality rate ranging from 30 to 60%. Currently, 30–50% of *C. auris* isolates are intrinsically resistant to amphotericin B. In this study, we characterized a clinical case of acquired amphotericin B resistance using whole-genome sequencing, a large-scale phenotypic screen, comprehensive sterol profiling, and genotypic reversion using CRISPR. Data obtained in this study provide evidence that a deletion resulting in a frameshift in *ERG3* significantly contributes to the observed resistant phenotype, and a nonsense mutation in *ERG4* may more modestly contribute to resistance. Characterization of this isolate also revealed that a fitness cost is associated with the abrogation of ergosterol production and its replacement with other late-stage sterols. This article presents a clinical case description of amphotericin B resistance from a frameshift mutation in *ERG3* in *C. auris* and marks an advancement in the understanding of antifungal resistance in this fungal pathogen.

## INTRODUCTION

In 2019, the Centers for Disease Control and Prevention (CDC) designated *Candidozyma auris* (previously known as *Candida auris*) as an urgent antimicrobial threat, making it the first fungal pathogen to be raised to this level of concern (1, 2). This is attributed to the multidrug-resistant characteristics of *C. auris*, its capacity to spread easily in health care facilities, and its potential to cause invasive candidiasis, especially among patients with weakened immune systems (3–6). Out of the three primary antifungal classes approved for the treatment of *Candida* infections, approximately 80–93% of *C. auris* isolates are resistant to fluconazole (triazole drug class), 35–50% display resistance to amphotericin B (polyene drug class), and 5–7% show resistance to the echinocandin antifungals (7–9).

Amphotericin B is frequently utilized due to its broad spectrum of activity and success in treating systemic fungal infections (10). First isolated from *Streptomyces nodosus* in 1953, amphotericin B disrupts fungal membrane permeability via ergosterol binding and has been observed to additionally enact oxidative damage (11, 12).

At the time of manuscript preparation, there were no clinical amphotericin B susceptibility breakpoints set forth by the Clinical Laboratory Standards Institute (CLSI) for *C. auris* (13), largely due to limited clinical outcome data and variability in *in vitro* minimum inhibitory concentration (MIC) determinations across testing methodologies. However, the CDC has established a tentative amphotericin B MIC breakpoint for *C. auris* at 2 mg/L. Though up to a third of *C. auris* isolates from the United States have MICs of 1 mg/L, caution is urged for *C. auris* amphotericin B MIC interpretation (6, 13–15).

Even though amphotericin B has been used in clinical practice since the 1950s, very few instances of drug resistance have been documented. Previously, acquired cases of amphotericin B resistance in *Candida* spp. have been shown to be associated with mutations in the genes encoding the ergosterol biosynthesis pathway (8). In *C. auris*, the only established mechanism of clinically acquired amphotericin B resistance is attributed to an indel in *ERG6* (16). Additionally, a nonsense mutation in *ERG3* has once been associated with amphotericin B resistance in a single clinical isolate but has yet to be tested/confirmed (17). Other mechanisms of amphotericin B resistance have been identified *in vitro*. Amphotericin B resistance can be induced by culturing *C. auris* in the presence of sub-lethal doses of amphotericin B (18). In other *Candida* spp., mutations in *ERG2*, *ERG3*, *ERG4*, *ERG5*, *ERG6*, and *ERG11* have been found to be associated with reduced susceptibility to amphotericin B (8, 10, 19–21).

Here, we present a case of acquired clinical amphotericin B resistance, which is attributed to mutations in the *C. auris ERG3* and *ERG4* genes, causing a premature stop codon in the C-5 sterol desaturase and delta (24 (24 (1)))-sterol reductase, respectively. We further investigated this isolate through a phenotypic screen, Cas9-mediated genetic repair, and sterol profiling. Our results support a causal link between the *ERG3* mutation and much of the amphotericin B resistance phenotype.

## MATERIALS AND METHODS

### Collection, Culturing, and Confirmation of Specimens

The first isolate (LNV001) from a patient was collected from the bronchial lavage in July 2022, while the second isolate (LNV002) from the same patient was collected from the urine in September 2022. Both samples were initially grown in Salt Sabouraud Dulcitol broth at 38°C at 250 rpm for 24–48 h. From culture, a 10 µL loop was used to streak culture on CHROMagar Candida (CHROMagar, Paris, France), where it was grown for 24–48 h at 36°C. Plates displayed a pinkish purplish growth and were confirmed as *C. auris* by matrix-assisted laser desorption ionization–time-of-flight mass spectrometry with reference library MALDI Biotyper CA library (version 2022) (Bruker, Billerica, MA).

### DNA extraction

Clinical isolates underwent bead-beating (FastPrep-24, MP Biomedicals, Irvine, CA) for four cycles at 6.0 m/s for 30 s with 5-min pauses in between. Then, genomic DNA (gDNA) from isolates was isolated using the PureFood Pathogen Kit on the Maxwell RSC (Promega, Madison, WI) per manufacturer’s protocol.

### Library prep and whole-genome sequencing

Extracted gDNA was library-prepped using DNA Prep Kit (Illumina, San Diego, CA) per manufacturer’s recommended protocol utilizing a STARlet automated liquid handler (Hamilton Company, Reno, NV). Paired-end sequencing (2 × 151 bp) was performed using NovaSeq 6000 (Illumina, San Diego, CA) with a read depth of 94× and 123× and a genome length of 12,288,577 and 12,267,689 for isolates LNV001 (SRR23958537) and LNV002 (SRR23109153), respectively.

### Bioinformatic analysis

The open-source software TheiaEuk was used to perform the *de novo* assembly, quality assessment, and genomic characterization of fungal genomes (22). Using the generated FASTA files, species taxon identification and clade typing were performed by Genomic Approximation Method for Bacterial Identification and Tracking (GAMBIT) (23). kSNP3 identified core genome single nucleotide polymorphisms (SNPs) between the two isolates, with confirmation and gene identification performed by Snippy to the *C. auris* clade III reference strain B11221 (GCA_0022775015.1) (24, 25).

### Phenotypic screens

#### Growth curve

*C. auris* isolates were grown on CHROMagar plates at 36°C for 48 h, and then, a single colony was isolated and regrown for purity for another 48 h at 36°C. The high-throughput phenotypic screening was performed utilizing Biolog PM1 and PM2a phenotypic microarray plates (Biolog, Inc., Hayward, CA) per manufacturer’s protocol for *Saccharomyces cerevisiae*. Absorbance was taken every 6–8 h for 72 h. Graphs were designed in R studio using the growthcurver package (26). Significance was determined by a student’s *t*-test of growth rate with the Bonferroni method to adjust for a large data set.

#### Dilution Spot

**Fig 1:**
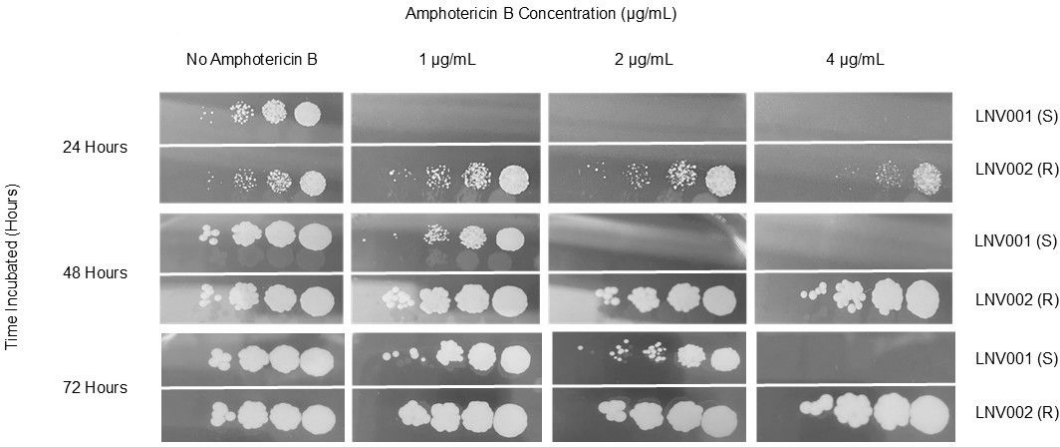
Dilution spot assay of amphotericin B-susceptible and resistant *C. auris* isolates. A ten-fold dilution series of LNV001 and LNV002 were plated on RPMI agar supplemented with varying concentrations of amphotericin B, and reading was observed every 24 h.

Single *C. auris* colonies from respective isolates were grown on CHROMagar plates for mass growth at 36°C for 48 h. Both isolates were standardized by absorbance in RPMI broth. A ten-fold dilution series was performed to gain a final dilution of 10^−4^. Dilution spots were plated on RPMI agar and checked every 24 h for 72 h. In Fig. 1, the lines in the background of each image are the overhead lights.

### Sterol analysis

Overnight cultures of *C. auris* strains were used to inoculate 20 mL MOPS-buffered RPMI to 0.1 OD_590_. Cultures were grown for 18 h at 35°C, 170 rpm. Cells were then harvested and split into two samples to enable the determination of dry weight and extraction of sterols. An internal standard of 10 µg of cholestanol was added to each sample, and sterols were extracted as previously described (27). Briefly, lipids were saponified using alcoholic KOH, and non-saponifiable lipids were extracted with hexane. Dried samples were derivatized by the addition of 0.1 mL BSTFA TMCS (99:1, Sigma) and 0.3 mL anhydrous pyridine (Sigma) and heating at 80°C for 2 h. TMS-derivatized sterols were analyzed and identified using gas chromatography–mass spectrometry (GC/MS) (Thermo 1300 GC coupled to a Thermo ISQ mass spectrometer, Thermo Scientific) and Xcalibur software (Thermo Scientific). The retention times and fragmentation spectra for known standards were used to identify sterols. Sterol composition was calculated from peak areas, as a mean of three replicates, and the relative quantity of sterols present was determined using a standard curve of the internal standard (cholestanol) and lanosterol and the dry weight of the samples.

### Episomal Plasmid-Induced Cas9 (EPIC)-mediated ERG3 and ERG4 modification in *C. auris* isolate LNV002

#### EPIC components

EPIC-mediated transformation of *C. auris* was performed as previously described (28). Briefly, guide sequence primers targeting *ERG3* and *ERG4* were ligated into pJMR19 following LguI (Thermo Scientific) digestion as previously described. Transformation repair templates were amplified from gBlock sequences (Integrated DNA Technologies) by PCR using Phusion Green Master Mix per manufacturer’s instructions (Thermo Scientific, Waltham, MA, USA), followed by subsequent QIAquick PCR purification (Qiagen). All strains, primers, and templates are listed in Table S1.

#### *C. auris* transformation

*C. auris* was cultured overnight to an OD_600_ of 1.8–2.2, and transformation reactions were assembled with salmon sperm DNA (Invitrogen), pJMR19, repair template DNA, and TE-LiAC + 55% PEG. EPIC-positive transformants were selected by growth on nourseothricin (200 mg/L)-supplemented YPD agar plates (28). Single colonies were patched onto YPD for screening. DNA isolation was achieved via treatment with a DNA extraction buffer consisting of 10 mM Tris pH 8.0, 2 mM EDTA, 0.2% Triton X-100, and 200 µg/mL Proteinase K. *ERG3* and *ERG4* PCR amplification and subsequent Sanger sequencing (Hartwell Center, St. Jude Children’s Research Hospital) were used to screen positive transformants. Sequenced transformants were then replica plated for plasmid ejection as previously described, and proper *ERG3* and *ERG4* sequence retention was confirmed with a second Sanger sequencing run (16, 29).

### Antifungal susceptibility testing

MICs for amphotericin B (Sigma-Aldrich) were determined by broth microdilution (BMD) in accordance with the M27-A4 from the CLSI, with considerations recommended by the CDC (15, 30). Amphotericin B MIC by Etest (bioMérieux, Marcy-l'Étoile, France) was determined per manufacturer’s instructions with CDC recommendations implemented. All susceptibility testing was performed in biological triplicate and determined visually for growth inhibition at 24 h.

## RESULTS

### Patient case description

**Fig 2:**
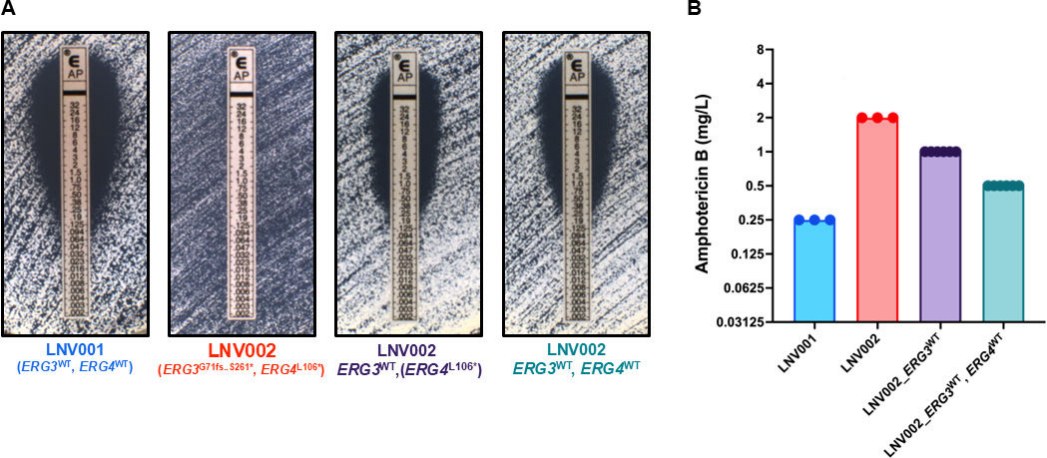
Amphotericin B MICs following *ERG3* and *ERG4* reversion to wild type. (**A**) Representative images of amphotericin B MICs at 24 h as determined by Etest (bioMérieux). (**B**) MICs determined by BMD in accordance with CLSI susceptibility testing. MICs were read visually for 100% growth inhibition at 24 h. Bars represent the modal MIC with points plotted for three biological replicates for each isolate and independent strain with MIC values for two independently derived LNV002_*ERG3*^WT^ and LNV002_*ERG3*^WT^, *ERG4*^WT^ strains shown.

Consecutive *C. auris* isolates were collected over a 2-month span in the summer of 2022 from a 76-year-old female. These two isolates were collected from the bronchial lavage and urine samples of the patient, respectively (Table 1). The initial isolate, LNV001, presented with an MIC value of 0.19 mg/L, yielding susceptibility to amphotericin B, while the latter isolate, LNV002, presented with a >32 mg/L MIC value, yielding high resistance to amphotericin B (Fig. 1 and 2). MIC values were determined by the Etest.

**TABLE 1:** Case study of acquired amphotericin B resistance in *C. auris^a^*

Isolate ID	Collection date	Isolation site	Amphotericin B MIC (mg/L)	Number of coding mutations
LNV001	July 2022	Bronchial lavage	0.19	9
LNV002	September 2022	Urine	>32	

^*a*^ *C. auris *isolates were collected over a 2-month span. Whole-genome sequencing was performed on both isolates, revealing nine sequence differences that result in coding changes between the genomes. MIC values determined by Etest.

### Whole-genome sequencing

The two isolates underwent paired-end sequencing on the NovaSeq 6000. Sequences were *de novo* assembled and quality checked by the bioinformatic workflow TheiaEuk (22). Confirmation of the respective isolates as *C. auris* was performed using GAMBIT, which further described each isolate as a member of Clade III (23). The sequences of the two isolates were genetically discordant from each other by nine sequence differences in coding regions determined by the bioinformatic workflow Snippy (Table 2) (25). Both isolates were resistant to fluconazole with an MIC value of >256 mg/L, and each was found to possess a mutation encoding the VF125AL amino acid substitution in sterol 14α-demethylase encoded by *ERG11,* which has been previously established to confer clinical fluconazole resistance and has exclusively been observed in Clade III isolates (7, 8). When these two isolates were compared with the Clade III reference isolate, LNV001 and LNV002 were 35 and 41 SNPs different in the core genome. Between the amphotericin B-susceptible and resistant isolates, the mutations of greatest interest to public health are the 10 bp deletion causing a frameshift at amino acid 71, leading to a premature stop codon at amino acid 132 in the C-5 sterol desaturase gene, *ERG3*, and the nonsense mutation causing a premature stop codon at amino acid 106 in the delta (24 (24 (1)))-sterol reductase gene, *ERG4*. Since amphotericin B is hypothesized to exert antifungal activity through direct interaction with ergosterol, we focused on *ERG3* and *ERG4* of the ergosterol biosynthesis pathway. When comparing the *ERG3* allele to a group of 44 isolates across Clades I–VI, with 25 of the isolates presenting with MIC values at or slightly above the amphotericin B breakpoint, we found that none of these isolates had an *ERG3* allele different from the respective clade reference strain (31).

**TABLE 2:** SNP differences between LNV001 and LNV002 in coding regions^*a*^

Protein (gene)	Predicted ortholog	Gene locus	Mutation	Mutation type
**C-5 sterol desaturase** (*ERG3*)	**C-5 sterol desaturase** (*ERG3*)	**CJI97_003811**	G71fs - > P132*	**Frameshift/stop**
**Delta (24 (24(1)))-sterol reductase** (*ERG4*)	**Delta (24 (24(1)))-sterol reductase** (*ERG4*)	**CJI97_002908**	L106*	**Stop**
1-phosphatidylinositol 4-kinase	1-phosphatidylinositol 4-kinase	CJI97_001150	N126D	Missense
Retromer subunit VPS35	Retromer subunit VPS35	CJI97_003028	I175V	Missense
Uncharacterized protein	Major Facilitator Superfamily	CJI97_000798	F232S	Missense
Uncharacterized protein	Mediator of RNA polymerase II transcription subunit 5	CJI97_002146	F275fs	Frameshift
Uncharacterized protein	Selenoprotein O	CJI97_002594	R243fs	Frameshift
Uncharacterized protein	*STE3*	CJI97_005168	A178V	Missense
Uncharacterized protein	Transcriptional regulator *ADR1*	CJI97_002218	E41Q	Missense

^*a*^ Nine coding regions have been identified to possess mutations from the former susceptible isolate. *ERG3* and *ERG4,* which are hypothesized to be responsible for the resistant phenotype, are bolded. Gene loci are from the clade III reference strain B11221. The “*” symbol designates a stop codon. SNP differences selected for further evaluation in this work are shown in shaded rows.

### EPIC-mediated reversion of ERG3 and ERG4 to wild type

To identify the influence of each mutated gene on the amphotericin B resistance, we utilized the EPIC genetic manipulation system to revert the Erg3 and Erg4 sequences to wild type (matching LNV001 and the B11221 reference) in LNV002 (28). While we were able to generate two independent *ERG3*^WT^ single reversions in LNV002, we were unable to generate *ERG4*^WT^ single reversions in LNV002 after multiple transformations. However, once *ERG3* had been reverted to wild type, we were able to generate two independent *ERG3*^WT^ and *ERG4*^WT^ double correction strains. When testing amphotericin B susceptibility using the diffusion test strip (Etest, bioMérieux) method, as recommended by the CDC, the *ERG3*^WT^ reversion in LNV002 resulted in dramatically increased amphotericin B susceptibility with an MIC shift from >32 mg/L to 0.38 mg/L (Fig. 2A). However, the additional reversion of *ERG4* to the wild-type allele (in strain LNV002_*ERG3*^WT^, *ERG4*^WT^) did not further increase amphotericin B susceptibility. By comparison, when testing amphotericin B MIC using the BMD method, reversion of the *ERG3* gene to the wild-type sequence resulted in a more modest decrease in amphotericin B MIC, and correction of both *ERG3* and *ERG4* to the wild-type allele resulted in a further one-dilution decrease in amphotericin B MIC (Fig. 2B).

### Absence of Ergosterol

We sought to determine if LNV002 produced ergosterol via sterol profiling because of the predicted interaction between amphotericin B and ergosterol. TMS-derivatized sterols in biological triplicate were analyzed and identified using GC/MS and Xcalibur software. The sterol profile of the susceptible LNV001 isolate largely consists of ergosterol and lanosterol (Table 3; Fig. S1). The sterol profile of the resistant isolate (LNV002) is mainly composed of ergosta-7,22,24 (28)-trienol, episterol, and lanosterol. Ergosterol was not detectable in the resistant isolate. LNV002_*ERG3*^WT^ (LNV002 *ERG3*c 39B; CRISPR repaired to *ERG3*^WT^) has a sterol profile that largely consisted of ergosta-5,7,22,24 (28)-tetraenol, lanosterol, and 14-methyl fecosterol, with a notable absence of detectable ergosterol. However, LNV002_*ERG3*^WT^, *ERG4*^WT^ (LNV002 *ERG3*c 39B *ERG4*c 7A; CRISPR repaired to *ERG3*^WT^ and *ERG4*^WT^) is mainly composed of ergosterol and lanosterol.

**TABLE 3:** Sterol composition of clinical and CRISPR-repaired *C. auris* isolates^*a*^

	Percentage of total sterol							
	LNV001(S)		LNV002(R)		LNV002 *ERG3*c 39B		LNV002 *ERG3*c 39B *ERG4*c 7A	
Sterol	Mean	±st.dev.	Mean	±st.dev.	Mean	±st.dev.	Mean	±st.dev.
Ergosta-5,8,22,24(28)-tetraenol	**0.3**	0.1					**0.8**	0.6
Ergosta-5,8,22-trienol	**0.1**	0.0			**0.6**	0.2	**0.4**	0.1
Ergosta-8,22,24(28)-trienol			**1.5**	0.0				
Zymosterol	**0.3**	0.0					**0.3**	0.2
Ergosterol	**45.2**	6.4					**62.8**	2.8
Ergosta-7,22-dienol	**0.6**	0.1					**0.6**	0.1
Unidentified			**1.1**	0.1				
Ergosta-5,7,22,24(28)-tetraenol					**49.2**	3.1	**1.6**	0.2
4,14-Dimethyl zymosterol	**0.3**	0.1	**2.8**	1.0			**1.1**	0.1
Fecosterol (ergosta-8,24(28)-dienol)			**0.4**	0.3				
Ergosta-7,22,24(28)-trienol			**53.7**	8.6				
14-Methyl fecosterol	**2.0**	0.5	**3.2**	1.0	**11.1**	0.5	**4.9**	0.4
Ergosta-5,7-dienol	**5.7**	7.6					**3.8**	0.4
Unidentified			**0.6**	0.1				
Episterol (ergosta-7,24(28)-dienol)			**17.3**	4.8	**0.5**	0.1	**0.4**	0.1
Ergosta-7-enol	**0.3**	0.2					**0.3**	0.1
4,4-Dimethyl-ergosta 8,14,24(28)-trienol	**0.2**	0.0	**0.1**	0.0				
14-Methyl ergosta-8,24(28)-dien-3-6-diol	**0.2**	0.0			**1.0**	0.1	**1.1**	0.1
Lanosterol	**39.7**	1.3	**17.3**	2.1	**34.2**	3.5	**19.9**	2.0
4-Methyl ergosta-8,24(28)-dienol	**0.7**	0.5						
4,4-Dimethyl cholesta-8,24-dienol	**1.4**	0.7	**0.8**	0.2				
4,4-Dimethyl cholesta-8,14,24-trienol			**0.3**	0.1				
Eburicol	**3.0**	0.5	**0.9**	0.3	**3.4**	0.3	**1.9**	0.3
Total	100.0		100.0		100.0		100.0	

	Dry weight content (µg/mL)							
	LNV001(S)		LNV002(R)		LNV002 *ERG3*c 39B		LNV002 *ERG3*c 39B *ERG4*c 7A	
	Mean	±st.dev.	Mean	±st.dev.	Mean	±st.dev.	Mean	±st.dev.
Lanosterol	**3.48**	1.23	**2.43**	0.51	**2.99**	0.46	**2.30**	0.69

^*a*^ TMS-derivatized sterols were analyzed and identified using GC/MS and XCALIBUR software. Sterols that mainly make up each isolate are bolded. Mean values are shown in shaded columns. Sterol compositions are out of 100%. Dry weight content of each strain is directly below the percentage of sterol table.

### Fitness cost associated with amphotericin B non-susceptibility

**Fig 3:**
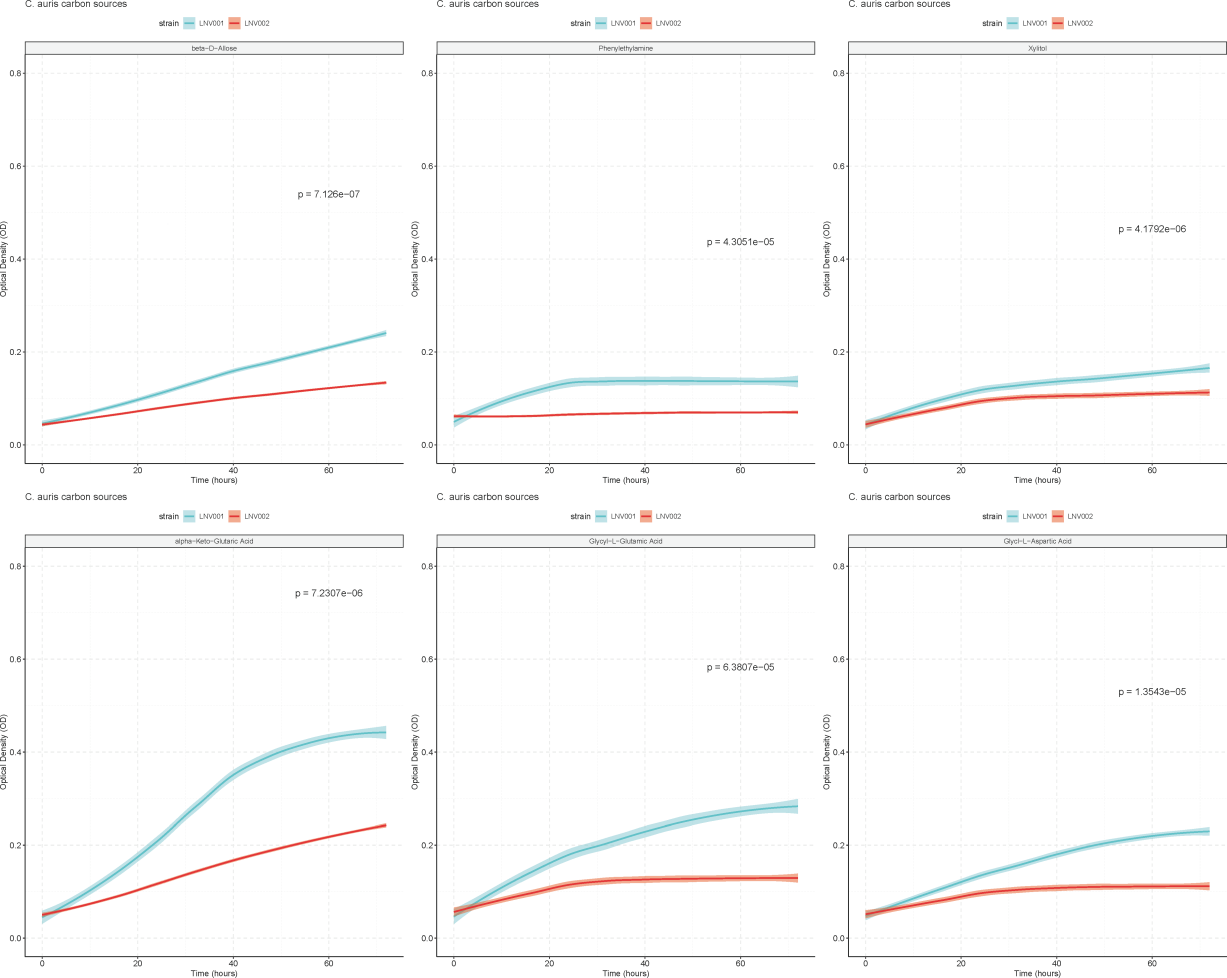
Significant growth of amphotericin B-susceptible isolate compared with resistant isolate Biolog Phenotypic plates PM1 and PM2a were inoculated with LNV001 and LNV002. Optical density was measured every 6–8 h for 72 h. The blue line represents LNV001, and the red line represents LNV002. Standard deviation is denoted by the blurring surrounding the lines. Significance was determined by a student’s *t*-test with the Bonferroni correction applied (α = 0.000263).

Amphotericin B resistance has been observed rarely for most *Candida* species (32). Evidence suggests that this is due to severe fitness defects caused by mutations in the ergosterol biosynthesis pathway (33). Since LNV002 had mutations in the ergosterol biosynthesis pathway, we hypothesized that there could be a fitness cost associated with *ERG3* and *ERG4* mutations in *C. auris*. To test this, we performed growth assays over 72 h on 190 different carbon sources. We determined fitness differences between LNV001 and LNV002, as described in the materials and methods (Table S2). Fourteen carbon sources were determined to be statistically significant for fitness defects in isolate LNV002. Carbon sources β-D-allose, phenylethylamine, xylitol, α-keto-glutaric acid, glycyl-L-glutamic acid, and glycyl-L-aspartic acid are associated with the highest fitness defects (Fig. 3). In general, this experiment concluded that there is a fitness cost associated with the nine coding mutations present in the resistant isolate when utilizing certain carbon sources for energy.

## DISCUSSION

### Amphotericin B is critical to managing this public health threat

Over 90% of *C. auris* clinical isolates demonstrate resistance to fluconazole, increasing our dependence on echinocandins and amphotericin B as the recommended frontline therapy for the treatment of invasive candidiasis and infections caused by *C. auris* specifically (15, 34). In 2021, the CDC noted that the number of echinocandin-resistant *C. auris* infections has tripled since 2020, leaving amphotericin B as a last resort to potentially treat these cases (3). Even though it has been observed that approximately 30% of *C. auris* isolates are resistant to amphotericin B, this agent remains a crucial drug in the antifungal armamentarium due to its broad-spectrum antifungal activity (10). *C. auris* is an emerging public health threat, and understanding its genetic basis of resistance is crucial for effective infection prevention and control.

### Interpretation of CRISPR results

From a clinical case, we isolated a pair of patient isolates, one susceptible and one resistant to amphotericin B collected within 2 months of each other. Between both isolates, a narrow list of non-synonymous mutations was revealed. Due to the involvement of ergosterol in the mechanism of action of amphotericin B, we focused our efforts on mutations present in genes, which are predicted to play a role in the ergosterol biosynthesis pathway, *ERG3* and *ERG4*.

Correction of the *ERG3* frameshift mutation (*ERG3*^G71fs..P132*^) in LNV002 restored clinical amphotericin B susceptibility, thus demonstrating a documented case of acquired amphotericin B resistance resulting from a deleterious *ERG3* mutation in a clinical *C. auris* isolate. The restored LNV002_*ERG3*^WT^ strain was found to possess an MIC value below the current determination of clinical resistance set by the CDC of 2 mg/L. When *ERG3* and *ERG4* were both restored to the wild-type sequence in LNV002, the MICs remained relatively unchanged from that of the individual LNV002_*ERG3*^WT^ strain, leaving only a small susceptibility difference persisting between LNV002_*ERG3*^WT^*, ERG4*^WT^ and that of LNV001. It remains possible that one of the other identified mutations observed in LNV002 may modestly contribute to amphotericin B resistance and account for the 0.5–1-dilution difference in MIC not attributable to mutations in *ERG3* or *ERG4*. One mutation of note that we speculate as a potential gene modifier candidate is gene locus CJI97_002218, an ortholog to *ADR1*, a transcriptional regulator in *C. albicans*. It has been found that *ADR1* has undergone transcriptional rewiring from *S. cerevisiae,* where now it directs the regulation of the ergosterol biosynthesis pathway in *C. albicans* (35). It is plausible that *ADR1* in *C. auris* could also contribute to the regulation of the ergosterol biosynthesis pathway, making this missense mutation a primary candidate for the difference in MIC between the LNV002_*ERG3*^WT^*, ERG4*^WT^ corrected strains and the susceptible isolate LNV001.

Additionally, it is notable that amphotericin B MIC values for the isolates and strains in this study varied by the susceptibility testing method used, particularly in the amphotericin B-resistant isolate LNV002 (>32 vs 2 mg/L). Similar variation in *C. auris* amphotericin B MIC values by testing method has previously been reported, with higher MIC values obtained when using diffusion test strips (36). Microbroth dilution determines MIC by complete inhibition of visible growth in RPMI broth, while Etest diffusion strips determine MIC by an ellipse on solid RPMI media and where visible fungal colonies contact the testing strip. The Etest methodology can be difficult to read and may be discerned subjectively. For example, while the LNV002 growth shown in Fig. 2 is small, actual inspection reveals that there are colonies contacting the testing strip up the gradient length above the 32 mark, and thus, the MIC is reported as >32 mg/L. Specifically for amphotericin B, the dynamic testing range for microbroth dilutions has been observed to be highly condensed, which contributes to challenges in determining clinical resistance in *C. auris,* where a large proportion of amphotericin B MICs are within one dilution of the CDC tentative clinical breakpoint. Nevertheless, both microbroth dilution and Etest diffusion strip-derived amphotericin B MIC determine LNV002 to exhibit clinical amphotericin B resistance and further demonstrated a consistent restoration of amphotericin B susceptibility upon *ERG3* mutation correction.

It is also notable that within the boundaries of our methods, the generation of an LNV002_*ERG4*^WT^-independent strain was not found to be possible. Therefore, we hypothesize that the *ERG3* mutation may have occurred prior to the *ERG4* mutation, with the subsequent deleterious *ERG4* mutation potentially compensating for fitness loss associated with amphotericin B resistance. Further elucidation of the role of *ERG4* in this process will require additional study.

### Replacement of ergosterol

In fungal cell membranes, ergosterol is a vital component responsible for membrane fluidity regulation, making the ergosterol biosynthesis pathway an attractive target for conventional antifungal use and drug development. In *C. albicans* and *S. cerevisiae,* the loss of function of lanosterol 14α-demethylase and C-5 sterol desaturase (*ERG11* and *ERG3*) has been associated with acquired amphotericin B resistance and for the exchange of ergosterol for other sterols such as lanosterol, eburicol, and 4,14-dimethyl-zymosterol in the cell membrane (37, 38). The resistant isolate described here does not produce ergosterol and showed enrichment of lanosterol, episterol, and late-stage sterols such as ergosta-7,22,24(28)-trienol. This altered membrane composition, which likely leads to amphotericin B resistance, is consistent with a loss of function of *ERG3* and *ERG4* based on their function in ergosterol biosynthesis. The loss of detectable ergosterol, while still uncommon, has been observed before in *C. auris*. In another amphotericin B-resistant isolate that had a premature stop codon in *ERG6*, ergosterol was notably absent, with cholesta-type sterols and early sterols comprising the sterol profile (16). We hypothesize that LNV002 is able to survive because late-stage sterols such as ergosta-7,22,24(28)-trienol and episterol are proposed to be able to stabilize the cell membrane in the absence of ergosterol. Additionally, the VF125AL amino acid substitution in lanosterol 14α-demethylase may be compensatory in nature, in order to support a truncated, non-functional C-5 sterol desaturase. However, determining the validity of this hypothesis is beyond the scope of this paper.

In the CRISPR-repaired isolates, LNV002_*ERG3*^WT^ and LNV002_*ERG3*^WT^, *ERG4*^WT^, susceptibility to amphotericin B, was restored. LNV002_*ERG3*^WT^ was mainly composed of the late-stage sterol Ergosta-5,7,22,24(28)-tetraenol with no detectable ergosterol. Since susceptibility is restored even though ergosterol is absent, amphotericin B seems to be able to interact with this late-stage sterol and exert its antifungal properties.

### Fitness cost identified with an amphotericin B-resistant isolate

Amphotericin B resistance is rarely observed for most *Candida* spp. This may be due to the fitness defects of mutations in the ergosterol biosynthesis pathway (33). In characterizing this resistant phenotype, a comparison between the susceptible and resistant isolates supplemented with 190 different carbon sources revealed a statistically significant fitness defect present on a few of these sources. These data may indicate a narrow fitness cost for combinatorial mutations in *ERG3* and *ERG4* in this isolate. Conversely, it is possible that among the other seven mutations detected by whole-genome sequencing, there are one or more modifiers that alleviate the fitness cost associated with perturbation of the ergosterol biosynthesis pathway. Furthermore, epigenetic mechanisms may contribute to amphotericin B resistance and potentially could compensate for this fitness cost.

### Conclusion

*C. auris* is spreading broadly, and the initially identified four major clades are no longer endemic solely to the area in which they were initially associated (39). The spread of this agent is resulting in extensive morbidity and mortality to patients in health care facilities across the world. Antifungal drug resistance will continue to evolve and spread unless we can identify and implement effective infection control mechanisms. With only three primary antifungal drug classes available to treat fungal infections in the United States and the multidrug-resistant characteristics of *C. auris,* the emphasis on anti-microbial stewardship, along with the development of novel antifungals, is more essential than ever to combat this pathogen.

This article characterizes a clinical case description of *C. auris* amphotericin B resistance resulting from a frameshift mutation in *ERG3*. In some outbreaks, one-third of *C. auris* isolates demonstrates amphotericin B resistance that is often attributed to an unidentified mechanism (9). This finding represents a significant advancement in understanding antifungal resistance in *C. auris,* and the knowledge generated can be used to design diagnostic tests to combat antifungal drug failure.

## SUPPLEMENTAL MATERIAL

Fig. 1 aac.00601-25-s0001.tif End of ergosterol biosynthesis pathway.

Supplemental material aac.00601-25-s0002.docx Captions for supplemental figure and tables.

Table S1 aac.00601-25-s0003.jpg Strains and primers from EPIC mediated ERG3 and ERG4 transformation.

Table S2 aac.00601-25-s0004.xlsx Growth curve assay across 190 carbon sources in LNV001 and LNV002.

Table S1 cont. aac.00601-25-s0005.jpg Strains and primers from EPIC mediated ERG3 and ERG4 transformation.

ASM does not own the copyrights to Supplemental Material that may be linked to, or accessed through, an article. The authors have granted ASM a non-exclusive, world-wide license to publish the Supplemental Material files. Please contact the corresponding author directly for reuse.

## References

[1] CDCgov. 2019. 2019 Antibiotic resistance threats report. CDC. https://www.cdc.gov/drugresistance/biggest-threats.html. 10.1097/CCM.0000000000004371 PMC7176261 32282351

[2] Liu F, Hu Z-D, Zhao X-M, Zhao W-N, Feng Z-X, Yurkov A, Alwasel S, Boekhout T, Bensch K, Hui F-L, Bai F-Y, Wang Q-M. 2024. Phylogenomic analysis of the Candida auris-Candida haemuli clade and related taxa in the Metschnikowiaceae, and proposal of thirteen new genera, fifty-five new combinations and nine new species. Persoonia 52:22–43. doi:10.3767/persoonia.2024.52.02 39161632 PMC11319837

[3] CDC. 2023. Increasing threat of spread of antimicrobial-resistant fungus in healthcare facilities. CDC online newsroom. CDC

[4] Spivak ES, Hanson KE. 2018. Candida auris: an emerging fungal pathogen. J Clin Microbiol 56:e01588-17. doi:10.1128/JCM.01588-17 29167291 PMC5786713

[5] Jeffery-Smith A, Taori SK, Schelenz S, Jeffery K, Johnson EM, Borman A, Manuel R, Brown CS, Candida auris Incident Management Team. 2018. Candida auris: an emerging fungal pathogen. Clin Microbiol Rev 31:e00029-17. doi:10.1128/CMR.00029-17 29142078 PMC5740969

[6] Jacobs SE, Jacobs JL, Dennis EK, Taimur S, Rana M, Patel D, Gitman M, Patel G, Schaefer S, Iyer K, Moon J, Adams V, Lerner P, Walsh TJ, Zhu Y, Anower MR, Vaidya MM, Chaturvedi S, Chaturvedi V. 2022. Candida auris pan-drug-resistant to four classes of antifungal agents. Antimicrob Agents Chemother 66:e0005322. doi:10.1128/aac.00053-22 35770999 PMC9295560

[7] Chow NA, Muñoz JF, Gade L, Berkow EL, Li X, Welsh RM, Forsberg K, Lockhart SR, Adam R, Alanio A, Alastruey-Izquierdo A, Althawadi S, Araúz AB, Ben-Ami R, Bharat A, Calvo B, Desnos-Ollivier M, Escandón P, Gardam D, Gunturu R, Heath CH, Kurzai O, Martin R, Litvintseva AP, Cuomo CA. 2020. Tracing the evolutionary history and global expansion of Candida auris using population genomic analyses. mBio 11:e03364-19. doi:10.1128/mBio.03364-19 32345637 PMC7188998

[8] Rybak JM, Cuomo CA, Rogers PD. 2022. The molecular and genetic basis of antifungal resistance in the emerging fungal pathogen Candida auris. Curr Opin Microbiol 70:102208. doi:10.1016/j.mib.2022.102208 36242897 PMC10364995

[9] Lockhart SR, Etienne KA, Vallabhaneni S, Farooqi J, Chowdhary A, Govender NP, Colombo AL, Calvo B, Cuomo CA, Desjardins CA, Berkow EL, Castanheira M, Magobo RE, Jabeen K, Asghar RJ, Meis JF, Jackson B, Chiller T, Litvintseva AP. 2017. Simultaneous emergence of multidrug-resistant Candida auris on 3 continents confirmed by whole-genome sequencing and epidemiological analyses. Clin Infect Dis 64:134–140. doi:10.1093/cid/ciw691 27988485 PMC5215215

[10] Carolus H, Pierson S, Lagrou K, Van Dijck P. 2020. Amphotericin B and other polyenes-discovery, clinical use, mode of action and drug resistance. J Fungi (Basel) 6:21. doi:10.3390/jof6040321 33261213 PMC7724567

[11] Dutcher JD. 1968. The discovery and development of amphotericin B. Dis Chest 54:Suppl. doi:10.1378/chest.54.supplement_1.296 4877964

[12] Mesa-Arango AC, Scorzoni L, Zaragoza O. 2012. It only takes one to do many jobs: amphotericin B as antifungal and immunomodulatory drug. Front Microbiol 3:286. doi:10.3389/fmicb.2012.00286 23024638 PMC3441194

[13] CLSI_LabNews. 2022. AST news update June 2022: hot topic. Available from: https://clsi.org/about/blog/ast-news-update-june-2022-hot-topic/

[14] Arendrup MC, Prakash A, Meletiadis J, Sharma C, Chowdhary A. 2017. Comparison of EUCAST and CLSI reference microdilution MICs of eight antifungal compounds for Candida auris and associated tentative epidemiological cutoff values. Antimicrob Agents Chemother 61:e00485-17. doi:10.1128/AAC.00485-17 28416539 PMC5444165

[15] CDC. 2023. Antifungal susceptibility testing and interpretation | Candida auris | fungal diseases. CDC. https://www.cdc.gov/fungal/candida-auris/c-auris-antifungal.html#print.

[16] Rybak JM, Barker KS, Muñoz JF, Parker JE, Ahmad S, Mokaddas E, Abdullah A, Elhagracy RS, Kelly SL, Cuomo CA, Rogers PD. 2022. In vivo emergence of high-level resistance during treatment reveals the first identified mechanism of amphotericin B resistance in Candida auris. Clin Microbiol Infect 28:838–843. doi:10.1016/j.cmi.2021.11.024 34915074 PMC9467277

[17] Ben Abid F, Salah H, Sundararaju S, Dalil L, Abdelwahab AH, Salameh S, Ibrahim EB, Almaslmani MA, Tang P, Perez-Lopez A, Tsui CKM. 2023. Molecular characterization of Candida auris outbreak isolates in Qatar from patients with COVID-19 reveals the emergence of isolates resistant to three classes of antifungal drugs. Clin Microbiol Infect 29:1083. doi:10.1016/j.cmi.2023.04.025 PMC10132836 37116861

[18] Carolus H, Pierson S, Muñoz JF, Subotić A, Cruz RB, Cuomo CA, Van Dijck P. 2021. Genome-wide analysis of experimentally evolved Candida auris reveals multiple novel mechanisms of multidrug resistance. mBio 12:e03333-20. doi:10.1128/mBio.03333-20 33820824 PMC8092288

[19] Kannan A, Asner SA, Trachsel E, Kelly S, Parker J, Sanglard D. 2019. Comparative genomics for the elucidation of multidrug resistance in Candida lusitaniae. mBio 10:e02512-19. doi:10.1128/mBio.02512-19 31874914 PMC6935856

[20] Ahmad S, Joseph L, Parker JE, Asadzadeh M, Kelly SL, Meis JF. 2019. ERG6 and ERG2 are major targets conferring reduced susceptibility to amphotericin B in clinical Candida glabrata isolates in Kuwait. Antimicrob Agents Chemother 63:e01900. doi:10.1128/AAC.01900-18 30455247 PMC6355561

[21] Rybak JM, Dickens CM, Parker JE, Caudle KE, Manigaba K, Whaley SG, Nishimoto AT, Luna-Tapia A, Roy S, Zhang Q, Barker KS, Palmer GE, Sutter TR, Homayouni R, Wiederhold NP, Kelly SL, Rogers PD. 2017. Loss of C-5 sterol desaturase activity results in increased resistance to azole and echinocandin antifungals in a clinical isolate of Candida parapsilosis. Antimicrob Agents Chemother 61:e00651-17. doi:10.1128/AAC.00651-17 28630186 PMC5571332

[22] Ambrosio FJ III, Scribner MR, Wright SM, Otieno JR, Doughty EL, Gorzalski A, Siao DD, Killian S, Hua C, Schneider E, Tran M, Varghese V, Libuit KG, Pandori M, Sevinsky JR, Hess D. 2023. TheiaEuk: a species-agnostic bioinformatics workflow for fungal genomic characterization. Front Public Health 11:1198213. doi:10.3389/fpubh.2023.1198213 37593727 PMC10428623

[23] Lumpe J, Gumbleton L, Gorzalski A, Libuit K, Varghese V, Lloyd T, Tadros F, Arsimendi T, Wagner E, Stephens C, Sevinsky J, Hess D, Pandori M. 2023. GAMBIT (genomic approximation method for bacterial identification and tracking): a methodology to rapidly leverage whole genome sequencing of bacterial isolates for clinical identification. PLoS One 18:e0277575. doi:10.1371/journal.pone.0277575 36795668 PMC9934365

[24] Gardner SN, Slezak T, Hall BG. 2015. kSNP3.0: SNP detection and phylogenetic analysis of genomes without genome alignment or reference genome. Bioinformatics 31:2877–2878. doi:10.1093/bioinformatics/btv271 25913206

[25] Seemann T. 2015. Snippy: rapid haploid variant calling and core genome alignment. https://github.com/tseemann/snippy.

[26] Sprouffske K, Wagner A. 2016. Growthcurver: an R package for obtaining interpretable metrics from microbial growth curves. BMC Bioinformatics 17:172. doi:10.1186/s12859-016-1016-7 27094401 PMC4837600

[27] Rybak JM, Xie J, Martin-Vicente A, Guruceaga X, Thorn HI, Nywening AV, Ge W, Souza ACO, Shetty AC, McCracken C, Bruno VM, Parker JE, Kelly SL, Snell HM, Cuomo CA, Rogers PD, Fortwendel JR. 2024. A secondary mechanism of action for triazole antifungals in Aspergillus fumigatus mediated by hmg1. Nat Commun 15:3642. doi:10.1038/s41467-024-48029-2 38684680 PMC11059170

[28] Doorley LA, Meza-Perez V, Jones SJ, Rybak JM. 2025. A candidozyma (Candida) auris-optimized episomal plasmid-induced Cas9-editing system reveals the direct impact of the S639F-encoding FKS1 mutation. J Infect Dis:jiaf285. doi:10.1093/infdis/jiaf285 PMC12455316 40569759

[29] Lombardi L, Oliveira-Pacheco J, Butler G. 2019. Plasmid-based CRISPR-Cas9 gene editing in multiple Candida species. mSphere 4:e00125-19. doi:10.1128/mSphere.00125-19 30867327 PMC6416365

[30] CLSI. 2017. Reference method for broth dilution antifungal susceptibility testing of yeasts. 4th ed. Clinical and Laboratory Standards Institute, Wayne, Pennsylvania, USA.

[31] Escandón P, Chow NA, Caceres DH, Gade L, Berkow EL, Armstrong P, Rivera S, Misas E, Duarte C, Moulton-Meissner H, Welsh RM, Parra C, Pescador LA, Villalobos N, Salcedo S, Berrio I, Varón C, Espinosa-Bode A, Lockhart SR, Jackson BR, Litvintseva AP, Beltran M, Chiller TM. 2019. Molecular epidemiology of Candida auris in Colombia reveals a highly related, countrywide colonization with regional patterns in amphotericin B resistance. Clin Infect Dis 68:15–21. doi:10.1093/cid/ciy411 29788045

[32] Arendrup MC, Patterson TF. 2017. Multidrug-resistant Candida: epidemiology, molecular mechanisms, and treatment. J Infect Dis 216:S445–S451. doi:10.1093/infdis/jix131 28911043

[33] Carolus H, Sofras D, Boccarella G, Sephton-Clark P, Biriukov V, Cauldron NC, Lobo Romero C, Vergauwen R, Yazdani S, Pierson S, Jacobs S, Vandecruys P, Wijnants S, Meis JF, Gabaldón T, van den Berg P, Rybak JM, Cuomo CA, Van Dijck P. 2024. Acquired amphotericin B resistance leads to fitness trade-offs that can be mitigated by compensatory evolution in Candida auris. Nat Microbiol 9:3304–3320. doi:10.1038/s41564-024-01854-z 39567662

[34] Pappas PG, Kauffman CA, Andes DR, Clancy CJ, Marr KA, Ostrosky-Zeichner L, Reboli AC, Schuster MG, Vazquez JA, Walsh TJ, Zaoutis TE, Sobel JD. 2016. Clinical practice guideline for the management of candidiasis: 2016 update by the infectious diseases society of America. Clin Infect Dis 62:e1–50. doi:10.1093/cid/civ933 26679628 PMC4725385

[35] Shrivastava M, Kouyoumdjian GS, Kirbizakis E, Ruiz D, Henry M, Vincent AT, Sellam A, Whiteway M. 2023. The Adr1 transcription factor directs regulation of the ergosterol pathway and azole resistance in Candida albicans. mBio 14:e0180723. doi:10.1128/mbio.01807-23 37791798 PMC10653825

[36] Arendrup MC, Lockhart SR, Wiederhold N. 2025. Candida auris MIC testing by EUCAST and clinical and laboratory standards institute broth microdilution, and gradient diffusion strips; to be or not to be amphotericin B resistant? Clin Microbiol Infect 31:108–112. doi:10.1016/j.cmi.2024.10.010 39426481 PMC11931498

[37] Vincent BM, Lancaster AK, Scherz-Shouval R, Whitesell L, Lindquist S. 2013. Fitness trade-offs restrict the evolution of resistance to amphotericin B. PLoS Biol 11:e1001692. doi:10.1371/journal.pbio.1001692 24204207 PMC3812114

[38] Sanglard D, Ischer FO, Parkinson T, Falconer D, Bille J. 2003. Candida albicans mutations in the ergosterol biosynthetic pathway and resistance to several antifungal agents. Antimicrob Agents Chemother 47:2404–2412. doi:10.1128/AAC.47.8.2404-2412.2003 12878497 PMC166068

[39] Rhodes J, Fisher MC. 2019. Global epidemiology of emerging Candida auris. Curr Opin Microbiol 52:84–89. doi:10.1016/j.mib.2019.05.008 31279224

