# Supplementary figures and images for: Acquired amphotericin B resistance attributed to a mutated ERG3 in Candidozyma auris
Source: Antimicrob Agents Chemother. 2025 Sep 22;69(11):e00601-25. doi: 10.1128/aac.00601-25 (PMC12587534; doi:10.1128/aac.00601-25)

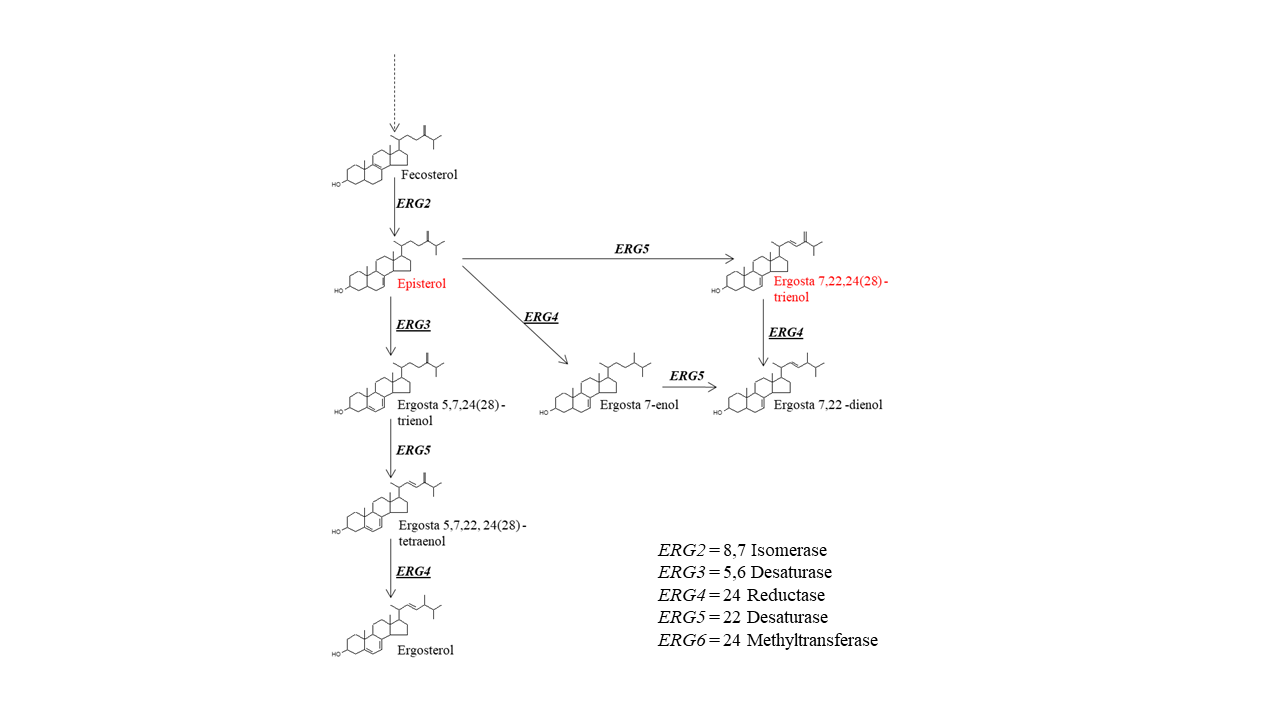

Supplement: Fig. 1 — End of ergosterol biosynthesis pathway. [file aac.00601-25-s0001.tif]

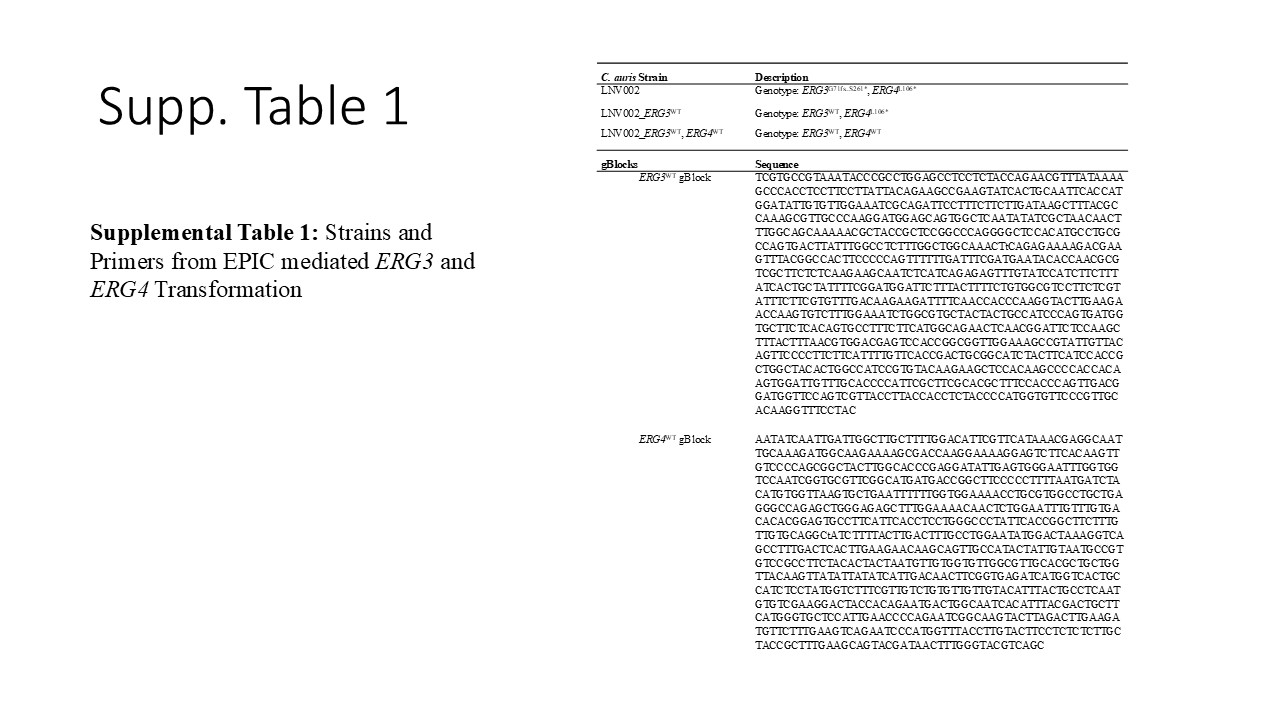

Supplement: Table S1 — Strains and primers from EPIC mediated ERG3 and ERG4 transformation. [file aac.00601-25-s0003.jpg]

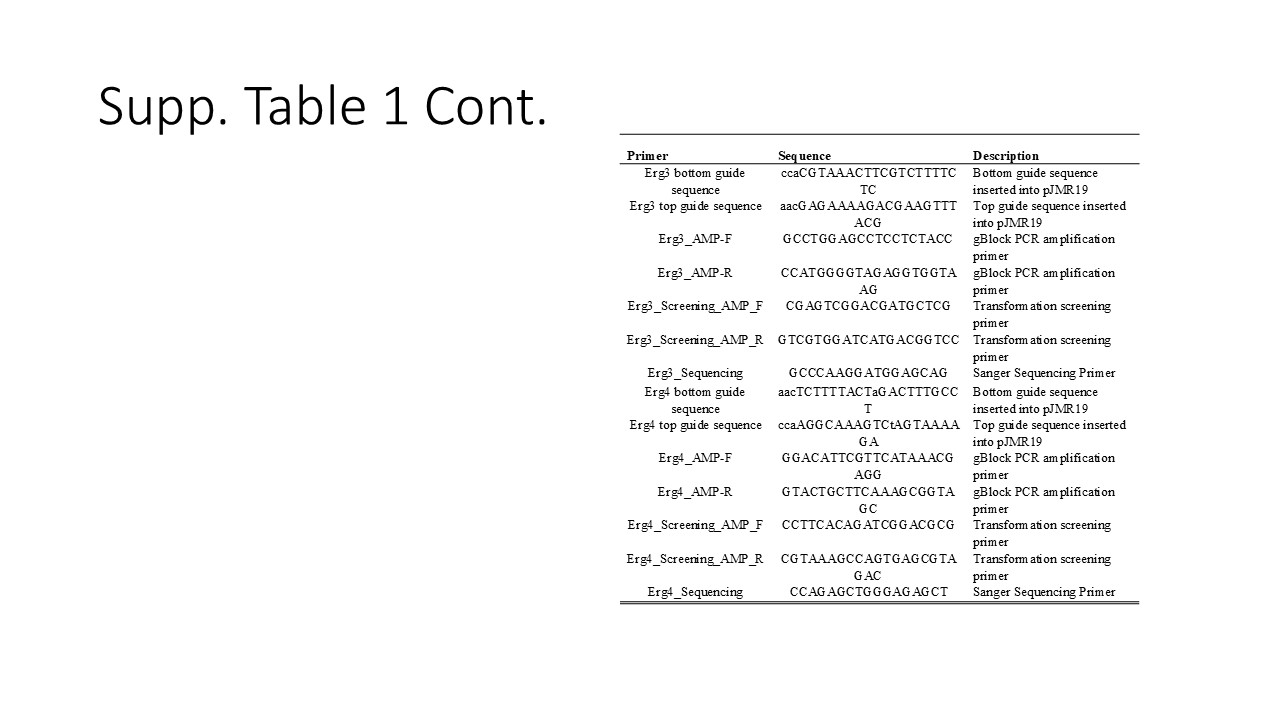

Supplement: Table S1 cont. — Strains and primers from EPIC mediated ERG3 and ERG4 transformation. [file aac.00601-25-s0005.jpg]
