# Supplementary material for: Acquired amphotericin B resistance attributed to a mutated ERG3 in Candidozyma auris
Source: Antimicrob Agents Chemother. 2025 Sep 22;69(11):e00601-25. doi: 10.1128/aac.00601-25 (PMC12587534; doi:10.1128/aac.00601-25)
Supplement: Supplemental material — Captions for supplemental figure and tables. [file aac.00601-25-s0002.docx]

Supplemental Figure 1

End of Ergosterol Biosynthesis Pathway: Frameshift/nonsense mutations in LNV002 are underlined. Sterols that compose LNV002 are red. The legend defines what enzyme the ERG genes code.

Supplemental Table 1

Strains and Primers from EPIC mediated ERG3 and ERG4 Transformation

Supplemental Table 2:

Growth Curve Assay Across 190 Carbon Sources in LNV001 and LNV002.
